# The associational pathway of emotional exhaustion among college students under perception of involution: the mediation model of self-compassion and psychological resilience

**DOI:** 10.3389/fpsyg.2026.1811047

**Published:** 2026-06-23

**Authors:** Si Li, Wenyan Chen

**Affiliations:** School of Marxism, Hainan College of Vocation and Technique, Haikou, Hainan, China

**Keywords:** emotional exhaustion, perception of involution, psychological resilience, self-compassion, university students

## Abstract

**Background:**

This study focuses on university students and examines the relationship between perception of involution and emotional exhaustion, with psychological resilience and self-compassion included as mediating variables.

**Methods:**

A cross-sectional design was employed. A survey was conducted among 763 university students via the Questionnaire Star platform.

**Results:**

Perception of involution was positively correlated with emotional exhaustion. Psychological resilience and self-compassion partially mediated the relationship between perception of involution and emotional exhaustion. Students with higher levels of perception of involution reported more severe emotional exhaustion and lower levels of psychological resilience and self-compassion, with the association between perception of involution and psychological resilience being particularly pronounced. Female students scored higher than males in perception of involution, emotional exhaustion, and psychological resilience, whereas male students scored higher in self-compassion.

**Conclusion:**

This study describes the associative patterns linking perception of involution with emotional exhaustion among university students and provides theoretical support for mental health education in higher education institutions. Targeted interventions may be considered according to differences in perception of involution, gender, and academic year to support the psychological well-being of university students.

## 1 Introduction

Emotional exhaustion (EE) originated from the concept of “job burnout” and refers to a state of physical and emotional depletion that occurs when individuals endure prolonged stress or face high-intensity emotional demands (Maslach and Jackson, 1981). According to the Conservation of Resources Theory (COR), individuals are prone to experiencing EE when they perceive their resources to be threatened, actually lose resources, or fail to obtain expected resource gains (Hobfoll, 1989). University life marks a critical stage in an individual's journey toward independence, during which students must adapt to new academic and social environments while confronting multiple challenges related to academics, interpersonal relationships, and future career development. These pressures are associated with a heavier psychological burden and greater susceptibility to EE. As early as 2021, researchers reported that nearly 40% of university students exhibited a high level of EE (Araoz, 2021). In recent years, the issue of EE among university students has garnered increasing scholarly attention.

Extensive empirical research has linked EE with multiple aspects of university students' psychological adjustment. Wei et al. (2022) found that students with higher EE tended to report poorer mental health status, while Feng and Dou (2024) reported that EE was associated with depressive symptoms and smartphone overuse. Related studies have also connected EE with depressive symptoms, self-efficacy, academic satisfaction, academic procrastination, sleep quality, and physical activity patterns (Conroy et al., 2022; Esteban et al., 2023; Jestin et al., 2023). Taken together, these findings suggest that EE should be considered within a broader framework involving mental health, academic adjustment, and health-related behaviors, rather than as an isolated psychological state. From the COR perspective, this broader pattern is consistent with the view that EE is closely related to students' perceived resource strain in academic and social contexts (Hobfoll, 1989).

### 1.1 Perception of involution and emotional exhaustion

Against the backdrop of increasingly scarce social resources and intensified competition, perception of involution (POI) has emerged as a significant psychosocial factor associated with the mental health of university students. The term “involution” was first introduced by anthropologist (Geertz 1963) to describe the stagnation in agricultural efficiency in Indonesia despite intensive labor. Later, sociologists extended this concept to denote “growth without development” (Huang, 2002), referring to a state where individuals face escalating competition under limited resources without achieving substantial breakthroughs. In contemporary society, involution often manifests as an irrational and involuntary form of excessive competition, where individuals are compelled to invest considerable time and energy in competing for scarce resources, ultimately receiving little return for their efforts, which is associated with severe emotional depletion and exhaustion (Yin et al., 2023). Thus, POI can be understood as students' subjective perception of competition, comparison, and resource strain in academic and social contexts.

Recent research has begun to examine the association between POI and EE within a broader resource-based and positive-psychology framework. POI has been shown to be directly associated with EE. For example, Ni et al. (2024) found that the academic involution atmosphere among university students was associated with higher levels of EE and poorer mental-health-related outcomes. Yan et al. (2022) further reported that involution-related behaviors were positively correlated with anxiety and stress, while Mulvey and Wright (2022) described irrational and involuntary competition as a context of persistent emotional resource strain. Taken together, these findings suggest that involution is not merely a sociocultural phenomenon, but a perceived competitive context closely related to students' emotional states. According to COR, individuals facing stress are motivated to acquire, maintain, and protect valuable resources (Hobfoll, 1989). In highly competitive and resource-anxious environments, continuous psychological resource investment may coexist with limited opportunities for resource restoration (Zhang et al., 2022). Positive psychology offers a complementary perspective by emphasizing positive psychological resources, such as psychological resilience (PR) and self-compassion (SC), that are closely related to students' adaptation in stressful contexts (Himmerich and Orcutt, 2021; Seligman and Csikszentmihalyi, 2000; Troy et al., 2023). Overall, POI, EE, PR, and SC can be understood as interrelated components of students' psychological adjustment in involution contexts, rather than as isolated constructs.

### 1.2 The potential mediating role of psychological resilience

PR was first proposed by American psychologist Anthony in the 1970s (Anthony and Koupernik, 1974), referring to an individual's positive adaptive capacity when facing adversity, stress, or challenges. As a positive personality trait closely associated with mental health, PR tends to be linked to greater capacity to cope with adverse external stimuli and to lower levels of stress- and setback-related negative experiences. In today's high-intensity academic and competitive environment, PR is widely regarded as a critical psychological resource and defense mechanism for university students to resist EE (Wang et al., 2021).

Prior empirical investigations have consistently indicated that PR is closely associated with lower levels of EE. For example, Ala et al. (2024) conducted a cross-sectional analysis with 526 university students and observed that individuals exhibiting stronger PR tend to experience lower levels of EE. Supporting this trend, Castillo-González et al. (2024) also affirmed a persistent inverse association between these two variables, supporting the buffering role of resilience in emotional depletion. In addition, PR has been linked to EE through its associations with negative emotions such as anxiety and depression (Chang et al., 2021; Li et al., 2024). These findings collectively emphasize the crucial role of PR in emotional regulation and the maintenance of mental health among university students. Meanwhile, POI, as a major psychosocial stressor faced by contemporary university students, is also closely linked to PR. According to the COR, resource-constrained and highly competitive involution contexts may be closely related to psychological resource depletion and EE. However, PR can serve as a core psychological resource that acts as a “buffer” in this process (Wang et al., 2021). It is associated with a more positive perspective on competitive pressure and stronger self-regulatory capabilities, which may be related to lower EE in the context of POI. Castillo-González et al. (2024), in a meta-analysis, further validated the protective function of PR by demonstrating a significant negative correlation between PR and job burnout. Furthermore, Ni et al. (2024), based on a survey of 1,150 Chinese university students, reported that the academic involution atmosphere was significantly associated with poorer students' mental health, with PR potentially showing a critical indirect role. This finding suggests that POI may be associated with EE both directly and indirectly through its relationship with individuals' PR.

### 1.3 The potential mediating role of self-compassion

SC theory posits that SC refers to the ability of individuals to treat themselves with understanding and kindness when facing failure or adversity (Neff, 2003). Its core components include self-kindness, common humanity, and mindfulness (Chen et al., 2022). For university students immersed in highly competitive and high-pressure involution environments, SC serves as a critical psychological resource that is associated with lower EE, less self-criticism, and fewer negative social comparisons in ongoing competitive contexts.

Evidence from earlier studies suggests that SC is significantly associated with lower EE. Those who demonstrate a greater degree of SC tend to report greater emotional acceptance and patience when facing difficulties and setbacks, along with lower emotional strain and better psychological wellbeing, including higher levels of life satisfaction (Kotera et al., 2022). For instance, Anjum et al. (2022) found that SC was associated with a weaker negative association between work alienation and EE. Among university students, SC is similarly regarded as an essential psychological resource that is associated with emotional regulation, lower psychological resource strain, and lower EE in the face of intense competition and pressure related to POI. Moreover, POI may be indirectly associated with EE through its relationship with individuals' SC levels. Sharma et al. (2025) found that SC was significantly negatively correlated with job burnout. Given the close association between job burnout and POI, university students entrenched in prolonged involution contexts may report intensified EE and psychological burnout in relation to lower SC. Additionally, students exposed to persistent high-pressure competition may report more self-criticism and negative social comparisons, which are negatively associated with SC (Neff, 2003). Supporting this, Chen et al. (2022) found through a questionnaire study that EE levels were in turn significantly associated with individuals' SC, providing empirical evidence for the serial pattern of associations between POI, lower SC, and higher EE.

### 1.4 Research objectives and hypotheses

Although POI has been recognized as a significant stressor associated with the mental health of university students (Yan et al., 2022), the pathways linking POI with EE have yet to be systematically explored. Existing studies on EE, involution, PR, and SC have provided valuable findings, but these findings are often discussed in separate lines of research. In particular, limited attention has been paid to the unique “resource dilemma arising from excessive competition” within involution contexts. Moreover, empirical support for group differences and mediating processes remains scarce. Therefore, integrating COR and positive psychology may help organize these variables within a more coherent theoretical framework and provide a synthesized basis for the present study. PR and SC, as key psychological resources, may mediate the relationship between POI and EE (Wang et al., 2021), but the specific associative pathways have not been fully validated.

Accordingly, drawing on the COR and the perspective of positive psychology, this study employs multi-group analysis to investigate the associative pathways linking POI with EE and to examine the indirect roles of PR and SC, with the aim of informing psychological interventions.

**Figure 1:**
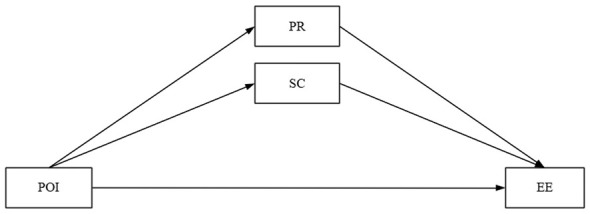
Theoretical hypothesized model. POI, Perception of involution; EE, Emotional exhaustion; SC, Self-compassion; PR, Psychological resilience.

Building on prior empirical evidence, this study develops a conceptual framework presented in Figure 1 and advances several testable propositions:

(1) POI is positively correlated with EE; (2) POI is negatively correlated with PR and SC; (3) PR and SC are negatively correlated with EE; (4) PR and SC partially mediate the relationship between POI and EE.

## 2 Materials and methods

### 2.1 Participants

Using a cross-sectional design and convenience sampling, this study surveyed students from several Chinese universities via Questionnaire Star (www.Sojump.com). Department heads helped introduce the survey and invite participation; all responses were voluntary and completed independently. Data collection was conducted between February and April 2025, and anonymity and confidentiality were strictly maintained. Using the 10:1 participant-to-item rule and allowing for 20% attrition, the 64-item questionnaire required a target sample of 768 participants (Kline, 2023). A total of 799 responses were obtained, followed by data screening based on the following criteria: questionnaires with duplicate IP addresses were removed to avoid repeated responses; completion-time outliers beyond three standard deviations were removed; and questionnaires in which the same response option was selected for over 70% of items within a single scale were eliminated to control for inattentive responding (Feng et al., 2024). This systematic screening process effectively ensured the reliability of the data and the accuracy of the research findings. Ultimately, 763 valid questionnaires were retained for analysis, including 384 male and 379 female participants (see Table 1). Before participating in the questionnaire, all individuals voluntarily provided written consent, which affirmed that the research process complied with ethical requirements and fully protected their personal rights, including confidentiality and independent decision-making.

**Table 1:** Profile of participants' demographic information.

Demographic characteristics	Category	Quantity	Percentage (%)	Correlation with the dependent variable
Gender	Male	384	50.3	0.111
	Female	379	49.7	
Age	18–20	168	22.0	−0.066
	20–22	178	23.3	
	22–24	189	24.8
	>24	228	29.9
Academic year	Freshman	184	24.1	0.053
	Sophomore	187	24.5	
	Junior	163	21.4	
	Senior	229	30.0	
Major category	Humanities	277	36.3	−0.067
	Social Sciences	329	43.1	
	Natural Sciences	157	20.6	

### 2.2 Measurement instruments

#### 2.2.1 Perception of involution (POI)

This study employed the POI scale developed by Zhang et al. (2024) to assess university students' levels of POI (e.g., “The people around me become outstanding through competition”). The scale consists of 18 items and uses a five-point Likert scale ranging from 1 (strongly disagree) to 5 (strongly agree) for self-assessment. Total scores range from 18 to 90, with higher scores indicating a greater perceived level of involution. Zhang et al. (2024) demonstrated that the scale has satisfactory structural validity and internal consistency reliability, making it suitable for evaluating the psychological health status of the Chinese population. In the present study, the Cronbach's α coefficient for the overall scale was 0.935.

#### 2.2.2 Emotional exhaustion questionnaire (EE)

To assess the extent of EE among college students, this study applied a standardized EE questionnaire. For example, students responded to items such as “I feel emotionally exhausted from study or work.” The instrument utilized a 7-point Likert-type scale, where 1 denoted “never” and 7 represented “always,” with greater scores reflecting more severe EE. Comprising three items in total, the aggregated scores ranged from 3 to 21. This instrument has been verified as appropriate for use among Chinese university samples and demonstrates robust applicability (Feng and Dou, 2024). In this research, the internal consistency of the EE scale, as measured by Cronbach's *alpha*, was 0.919.

#### 2.2.3 Connor-davidson resilience scale (CD-RISC)

To evaluate participants' PR, the CD-RISC was administered. A representative item includes “I can easily adjust to changes.” The instrument contains 25 items, each rated on a 5-point scale from 0 (“not true at all”) to 4 (“true nearly all the time”), yielding total scores between 0 and 100. Higher scores reflect greater resilience. The CD-RISC has been extensively employed in research involving university students and has shown strong psychometric properties in prior work (Chen X. Q. et al., 2024). In the current study, the Cronbach's α for this scale was calculated at 0.949.

#### 2.2.4 Self-compassion scale-short form (SCS-SF)

University students' SC was assessed using the short form of the SCS-SF. A sample item reads, “When I don't succeed at something important, I tend to feel frustrated with myself.” This instrument consists of 12 statements, each scored on a 5-point scale ranging from 1 (“almost never”) to 5 (“almost always”). Positively worded items are scored directly, while negatively framed items are reverse-coded before summation. The total scores span from 12 to 60, with higher values indicating greater levels of SC. The SCS-SF has been widely validated in research with Chinese student populations, and previous studies have confirmed its satisfactory internal consistency and structural validity (Chen T. Y. et al., 2024). In this investigation, the scale yielded a Cronbach's *alpha* of 0.962.

### 2.3 Statistical analyses

To evaluate the research hypotheses and build the proposed model, a sequential analytical strategy was applied. Following recommendations in Hair et al. (2021) and Kline (2023), we grounded the use of PLS-SEM in its suitability for prediction-oriented studies, handling complex latent variable structures, and accommodating mediation pathways in cross-sectional survey data. Path coefficient estimation and bootstrapping were employed to assess the significance and stability of relationships, consistent with established SEM practices. Independent sample *t*-tests and one-way ANOVA were conducted to examine group differences, as supported by standard statistical methodology for comparing subgroup means. Lastly, a multi-group PLS approach was utilized to assess structural variations across different groups, following the MICOM procedure to ensure measurement invariance.

## 3 Results

The results are presented in the following order: preliminary data screening, measurement model assessment, structural model assessment, mediation analysis, group differences, and multigroup analysis. Descriptive statistics, skewness, kurtosis, KMO values, and Bartlett's test results are summarized in Table 2. The skewness and kurtosis values were within the recommended ranges proposed by Kim (2013). In addition, all KMO values were above 0.5, and Bartlett's tests were statistically significant, indicating that the data were suitable for subsequent factor-based analyses.

**Table 2:** Summary of descriptive measures.

Constructs	*N*	MIN	MAX	M ±SD	SK	Kur	KMO	Bartlett
POI	763	12	60	32.29 ± 10.045	0.038	−0.086	0.780	< 0.001
EE	763	3	21	8.920 ± 3.352	0.950	2.732	0.750	< 0.001
SC	763	9	45	33.670 ± 8.454	−0.334	−0.183	0.819	< 0.001
PR	763	20	100	65.070 ± 15.359	0.714	−0.154	0.905	< 0.001

*N*, sample size; M ± SD, mean ± standard deviation; MIN, minimum value; MAX, maximum value; SK, skewness; Kur, kurtosis; KMO, Kaiser-Meyer-Olkin measure of sampling adequacy; *P*-values < 0.001 indicate that the variables are suitable for factor analysis; POI, Perception of involution; EE, Emotional exhaustion; SC, Self-compassion; PR, Psychological resilience.

### 3.1 SEM analysis

To evaluate both the measurement and structural components of the proposed model, this study adopted the partial least squares structural equation modeling (PLS-SEM) technique. This method was chosen for several methodological reasons. First, PLS-SEM is suitable for prediction-oriented research because it emphasizes explained variance and predictive relevance. Second, PLS-SEM is appropriate for estimating relatively complex models involving multiple latent variables, observed indicators, and indirect pathways. Third, PLS-SEM provides a flexible framework for bootstrapping-based significance testing of direct and indirect associations. In the present study, the main analytical focus was to examine the associative pathway linking POI, PR, SC, and EE, and to assess the explanatory and predictive performance of the model through path coefficients, R^2^, Q^2^, and bootstrapped indirect associations. These aims are consistent with the strengths of PLS-SEM. By contrast, covariance-based SEM (CB-SEM) is generally more appropriate when the primary goal is to confirm a well-established theoretical model and evaluate overall model fit based on the covariance matrix. Because the present study was conducted in the relatively underexplored context of perceived involution among university students and focused more on explained variance, predictive relevance, and mediation pathways than on confirming a mature covariance structure, PLS-SEM was considered more aligned with the study objectives. Considering that this research investigates the association between POI and EE in a university student population, and employs a model with four latent variables and 64 observed items across a sample of 763 respondents, PLS-SEM was deemed both statistically sound and practically efficient (Hair et al., 2021).

### 3.2 Measurement model

In accordance with the guidelines proposed by Hair et al. (2021), the current study conducted a comprehensive evaluation of the measurement properties of all scales, focusing on their reliability and validity. Regarding internal consistency, both indicator loadings and composite reliability (CR) were employed as assessment metrics. Item deletion was conducted based on a combined consideration of outer loadings, CR, AVE, and the theoretical coverage of each construct, rather than relying solely on a mechanical cut-off value. Following the guideline that loadings around or above 0.708 are generally preferred in PLS-SEM, items with clearly weak loadings were reviewed and removed when their deletion supported the reliability and convergent validity of the measurement model without weakening the conceptual meaning of the construct (Hair et al., 2021). Specifically, POI3, POI4, POI8, POI16, POI17, and POI18 were removed from the POI scale; SC10, SC11, and SC12 were removed from the SC scale; and PR14, PR15, PR21, PR24, and PR25 were removed from the PR scale. All three EE items were retained. Several retained indicators had loadings slightly below 0.708 but were close to the recommended value; they were retained because the corresponding constructs still met the recommended standards for CR and AVE, and their retention helped preserve the content coverage of the original scales. After refinement, the final item counts were as follows: 12 items remained for the POI scale, 9 for the SC scale, 20 for the PR scale, and all 3 items for the EE scale were retained. As displayed in Table 3, each construct achieved a Cronbach's alpha exceeding 0.7 and a CR value above 0.8, reflecting satisfactory reliability.

**Table 3:** Measurement model quality indicators.

Constructs	Items	Loadings	Cronbach' α	CR	AVE
POI	POI1	0.756	0.935	0.944	0.587
	POI2	0.833			
	POI5	0.701			
	POI6	0.729			
	POI7	0.756			
	POI9	0.701			
	POI10	0.729			
	POI11	0.792			
	POI12	0.783			
	POI13	0.841			
	POI14	0.709			
	POI15	0.841			
EE	EE1	0.917	0.919	0.949	0.860
	EE2	0.943			
	EE3	0.922			
SC	SC1	0.870	0.949	0.956	0.709
	SC2	0.862			
	SC3	0.858			
	SC4	0.883			
	SC5	0.854			
	SC6	0.818			
	SC7	0.809			
	SC8	0.803			
	SC9	0.816			
PR	PR1	0.730	0.962	0.965	0.581
	PR2	0.740			
	PR3	0.751			
	PR4	0.759			
	PR5	0.706			
	PR6	0.787			
	PR7	0.707			
	PR8	0.734			
	PR9	0.778			
	PR10	0.782			
	PR11	0.784			
	PR12	0.781			
	PR13	0.806			
	PR16	0.783			
	PR17	0.788			
	PR18	0.805			
	PR19	0.718			
	PR20	0.784			
	PR22	0.759			
	PR23	0.746			

POI, perception of involution; EE, emotional exhaustion; SC, self-compassion; PR, psychological resilience; CR, composite reliability; AVE, average variance extracted.

Construct validity was checked using AVE, HTMT, and Fornell-Larcker. As shown in Supplementary Tables 1, 2, AVE (>0.50), HTMT (< 0.85), and AVE-root comparisons all met the criteria, confirming adequate measurement quality.

### 3.3 Evaluation of common method bias

To examine the potential presence of common method variance, this study conducted Harman's one-factor analysis as recommended in prior literature. An unrotated exploratory factor analysis was performed, which yielded seven distinct factors with eigenvalues exceeding 1. The first unrotated factor accounted for 29.251% of the total variance, which is below the 40% threshold commonly cited as a benchmark for concern (Podsakoff and Organ, 1986). In addition to this statistical check, several procedural considerations were incorporated during questionnaire administration. Participation was voluntary, anonymity and confidentiality were emphasized, and respondents completed the questionnaire independently, which helped limit evaluation apprehension and socially desirable responding. Nevertheless, because all focal variables were measured using self-reported questionnaires at a single time point, common method bias cannot be completely ruled out. Therefore, the findings should be interpreted with appropriate caution.

### 3.4 Structural model

#### 3.4.1 Collinearity assessment

To examine multicollinearity in the structural model, the Variance Inflation Factor (VIF) was calculated for each relevant predictor. All VIF values were below the recommended threshold of 3.3, indicating no evident collinearity concern among the structural model variables (Hair et al., 2021).

#### 3.4.2 Evaluation of path effects in the structural framework

To assess the structural associations, bootstrapping with 5,000 resamples was performed within the PLS-SEM framework. The results showed statistically significant associations for all hypothesized paths. POI was positively associated with EE and negatively associated with SC and PR. SC and PR were both negatively associated with EE. The path coefficients, confidence intervals, *t*-values, and *p*-values are presented in Table 4. Regarding the magnitudes of the standardized path coefficients, the POI–EE association (β = 0.452) and the SC–EE association (β = −0.373) were the most prominent paths in the model. The associations of POI with PR (β = −0.253) and SC (β = −0.235) were more modest, whereas the PR–EE association (β = −0.082) was comparatively small. Therefore, the statistical significance of the hypothesized paths should be interpreted together with their relative magnitudes.

**Table 4:** Significance results of structural model relationships.

Relationships	Original sample (O)	2.5%	97.5%	*t*	*p*	Results
POI → SC	−0.235	−0.313	−0.155	5.823	< 0.001	Yes
POI → EE	0.452	0.388	0.515	13.96	< 0.001	Yes
POI → PR	−0.253	−0.343	−0.178	6.064	< 0.001	Yes
SC → EE	−0.373	−0.435	−0.307	11.387	< 0.001	Yes
PR → EE	−0.082	−0.154	−0.017	2.367	0.018	Yes

POI, perception of involution; EE, emotional exhaustion; SC, self-compassion; PR, psychological resilience.

In addition, the model accounted for 44.1% of the variance in EE (*R*^2^ = 0.441; adjusted *R*^2^ = 0.439), and the Q^2^ value was 0.678, indicating acceptable explanatory relevance for EE.

#### 3.4.3 Mediation effect analysis

**Figure 2:**
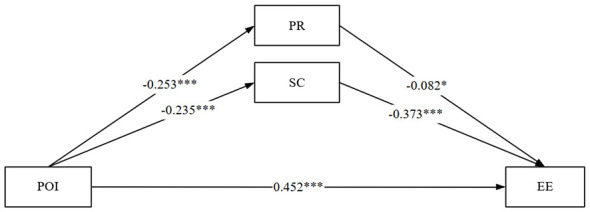
Research model. ^***^p < 0.001; ^*^p < 0.05.

Bootstrapping-based mediation analysis was conducted to examine the indirect associations from POI to EE through SC and PR. After controlling for gender, age, and academic year, the indirect association through SC was statistically significant. The indirect association through PR was also statistically significant. The direct association between POI and EE remained statistically significant, indicating partial mediation through SC and PR. The indirect association through SC (β = 0.088) was larger than the indirect association through PR (β = 0.021), suggesting that SC accounted for a larger portion of the indirect POI–EE association in the present sample. However, given the cross-sectional design, these indirect estimates should be understood as associative rather than causal evidence. These results are presented together with the structural path results in Table 5, and the final research model with standardized path coefficients is shown in Figure 2.

**Table 5:** Mediation analysis.

Relationship	Indirect effect	2.5%	97.5%	*t*	*p*	Mediation type
POI → SC → EE	0.088	0.056	0.122	5.124	< 0.001	PM
POI → PR → EE	0.021	0.004	0.044	2.039	0.042	PM

POI, perception of involution; EE, emotional exhaustion; SC, self-compassion; PR, psychological resilience; PM, partial mediation.

### 3.5 Group differences analysis

Group differences were examined using independent-sample t-tests and one-way ANOVA. Gender differences were statistically significant for POI, EE, SC, and PR. Female students reported higher mean scores for POI, EE, and PR, whereas male students reported higher mean scores for SC. Academic-year differences were also examined across the four main variables. The ANOVA results showed statistically significant differences in POI [*F* = 32.534, *df* = (3, 759), *p* < 0.001] and PR [*F* = 12.376, *df* = (3, 759), *p* < 0.001], whereas no statistically significant academic-year differences were observed for EE or SC (*p* > 0.05). Bonferroni *post-hoc* tests showed that 3rd-year students had lower POI scores than 1st-year, 2nd-year, and 4th-year students, while 4th-year students had higher POI scores than students in the other academic years. For PR, 1st-year students had higher scores than 2nd-year, 3rd-year, and 4th-year students.

Participants were further divided into high- and low-POI groups according to their POI scores. The high-POI group reported higher EE scores, whereas the low-POI group reported higher SC and PR scores. The group-difference results are presented in Table 6.

**Table 6:** Group differences in main study variables.

Comparison	Construct	Group 1 M ±SD (N)	Group 2 M ±SD (N)	*t*	*P*
Gender	POI	Male 29.55 ± 8.780 (384)	Female 35.07 ± 10.485 (379)	−7.887	< 0.001
	EE	Male 8.55 ± 2.804 (384)	Female 9.30 ± 3.795 (379)	−3.080	0.002
	SC	Male 35.02 ± 7.606 (384)	Female 32.31 ± 9.041 (379)	4.486	< 0.001
	PR	Male 63.23 ± 14.184 (384)	Female 66.93 ± 16.272 (379)	−3.351	< 0.001
POI group	EE	Low POI 7.57 ± 2.861 (381)	High POI 10.27 ± 3.263 (382)	−12.177	< 0.001
	SC	Low POI 34.47 ± 6.970 (381)	High POI 32.88 ± 9.655 (382)	2.608	0.009
	PR	Low POI 68.82 ± 16.090 (381)	High POI 61.32 ± 13.617 (382)	6.952	< 0.001

POI, perception of involution; EE, emotional exhaustion; SC, self-compassion; PR, psychological resilience; *N*, sample size; *M*, mean; SD, standard deviation.

### 3.6 Multigroup analysis

Given the group differences observed in Section 3.5, multigroup PLS analysis was conducted to compare the structural associations between the high- and low-POI groups. Prior to comparing group-specific paths, construct equivalence was examined through MICOM. In Smart PLS 4.0, structural invariance was established by applying the same model specification and algorithm settings across groups. As shown in Supplementary Table 3, compositional invariance was supported for POI, SC, and PR, whereas EE did not meet the criterion. Therefore, the multigroup results were interpreted with caution. The path coefficient comparison is presented in Table 7. The POI–PR path differed significantly between the two groups. Specifically, the absolute magnitude of the POI–PR path was larger in the high-POI group than in the low-POI group, although the directions of the associations differed between groups: the association was negative in the high-POI group but positive in the low-POI group. From a magnitude perspective, this result suggests that the POI–PR association differed across POI levels. Because EE did not fully meet the MICOM criterion, this subgroup pattern should be interpreted cautiously.

**Table 7:** Differences in structural paths across POI levels.

Relationship	Original (High)	Original (Low)	Difference (High—Low)	*P*
POI → PR	−0.269	0.169	−0.438	0.018

POI, perception of involution; PR, psychological resilience.

## 4 Discussion

This research aimed to gain a deeper understanding of how PR and SC are related to the link between POI and EE. The central assumption of the study proposed that POI would show a positive association with EE, while PR and SC were expected to act as partial mediators in this relationship. The upcoming section will elaborate on the key findings, interpreting them in light of the initial research objectives and the hypotheses formulated at the outset.

### 4.1 The positive correlation between perception of involution and emotional exhaustion

This study found a significant positive correlation between university students' POI and EE, supporting Hypothesis 1. This result is consistent with the findings of Ni et al. (2024), who reported that the academic involution atmosphere was associated with greater meaningless competition and resource depletion, and with higher levels of psychological exhaustion. Further group analysis revealed that, among the high and low POI groups based on the scores, the high POI group exhibited significantly higher EE scores than the low POI group. This trend echoes related studies in the workplace domain. Dou et al. (2022) pointed out that repetitive tasks and peer competition in work environments, which are inefficient activities, were associated with EE in the context of the continuous depletion of individual resources. Although the study focused on employees, a similar pattern can be observed in university students' academic “involution”: for example, excessive time investment in pursuit of higher academic scores or passive participation in inefficient studying in the context of peer comparison, a pattern that may ultimately coincide with physical strain and EE. From the perspective of COR (Hobfoll, 1989), when individuals realize that their resources (such as time, energy, and social recognition) are continuously depleted in involutional competition without receiving appropriate compensation, this situation may correspond to intense stress responses and elevated EE (Zhang et al., 2022). Students in the high POI group in this study may have been in such a “resource investment-reward imbalance” state for an extended period, thus exhibiting higher levels of exhaustion. The findings of this study also validate the COR explanatory pathway, highlighting the potential threat of involutional competition to university students' mental health and providing practical insights for alleviating excessive competition in education and optimizing resource allocation.

### 4.2 The negative correlation between perception of involution and psychological resilience, self-compassion

The results of this study showed that university students' POI was significantly negatively correlated with PR, supporting Hypothesis 2. According to COR, PR can serve as a core psychological resource associated with less severe negative experiences related to POI (Wang et al., 2021). PR is associated with a more positive view of competitive pressures and with stronger self-regulatory abilities, and is linked to fewer negative feelings related to involution. This finding is consistent with the study by Castillo-González et al. (2024), which found a significant negative correlation between PR and job burnout, further confirming the protective role of PR. Group comparison results showed that the high POI group scored significantly lower on PR than the low POI group. Furthermore, multi-group analysis showed significant group variation in the POI–PR association, with a stronger absolute path coefficient in the high-POI group. This pattern is consistent with the view that students with higher POI may show a closer link between perceived involution and PR. In high-pressure academic contexts characterized by excessive competition and resource comparison, repetitive self-depletion and continuous anxiety may be accompanied by lower levels of positive mindset and self-regulation when students face stress (Yan et al., 2022). Additionally, ongoing high-intensity involution contexts may be associated with limited psychological space and fewer opportunities for students to restore their PR, which is consistent with difficulties in establishing effective stress-coping mechanisms.

This study also found that POI was negatively correlated with SC, supporting Hypothesis 2. Consistent with Sharma et al. (2025), a significant negative correlation between SC and job burnout was found, suggesting that university students in long-term involution contexts are more likely to experience EE and psychological burnout. Moreover, students in high-pressure competitive environments are more prone to self-criticism and negative social comparisons, which may relate to weaker development of SC (Neff, 2003). Group analysis revealed that the high POI group had significantly lower SC scores than the low POI group. Students in the high POI group are constantly immersed in an environment of high-intensity competition and excessive comparison, always worried about being surpassed. This constant pressure may be accompanied by self-criticism, substantial energy investment in compensating for shortcomings, and neglect of their emotional needs. Overemphasis on external standards and others' evaluations, frequent negative social comparisons, and an inability to accept their imperfect selves may correspond to weaker SC development, which aligns with significantly lower SC scores than the low POI group. These findings not only expand the role of SC in coping with involution stress but also provide new perspectives and theoretical support for the development of localized mental health education and intervention strategies.

### 4.3 The negative correlation between psychological resilience, self-compassion, and emotional exhaustion

This study found a significant negative correlation between PR and EE, which is consistent with Hypothesis 3. This finding aligns closely with existing research. Ala et al. (2024) indicated that PR was significantly negatively associated with EE, and Durmaz and Üsenmez (2024) further revealed a strong negative association between the two, suggesting that higher levels of PR are linked to greater capacity to cope with EE.. Additionally, a meta-analysis by Castillo-González et al. (2024) supports this conclusion, identifying PR as a key protective factor linked to less severe EE. According to COR, individuals instinctively protect their resources to cope with external pressures. When resources are abundant, individuals are more likely to maintain psychological stability; conversely, when resources are depleted, they are more likely to experience tension and exhaustion. As an internal resource, PR is associated with university students' coping with involution pressure through an optimistic mindset and effective coping strategies, as well as stronger resource management abilities and lower risk of EE. In contrast, students with lower levels of PR may report greater resource strain in continuous competition, which is associated with psychological fatigue. Therefore, in this study, a stable negative relationship between PR and EE was observed.

This study also found that SC was significantly negatively correlated with EE, further validating Hypothesis 3. This result is consistent with Anjum et al. (2022), confirming the protective mechanism of SC in the context of involution. Kotera et al. (2021) also found that SC was associated with lower EE under long-term stress, with strong stability. Further research has pointed out that individuals with higher levels of SC are more likely to approach setbacks with acceptance, a pattern linked to less emotional distress and greater well-being and life satisfaction (Kotera et al., 2022). According to Neff (2003) in the Self-Compassion Theory, the protective mechanism of SC may operate through three pathways: first, self-kindness may correspond to less self-blame after failure; second, a sense of common humanity may correspond to less feelings of isolation, such as “I am not as good as others”; third, mindfulness may correspond to lower EE in the context of ruminating on failure. For example, when facing academic pressure related to GPA, students with high SC are more likely to adopt a “failure is part of growth” mindset rather than falling into extreme thinking of “I must be perfect” (Anjum et al., 2022). This study further verifies that SC can effectively break the “self-criticism—anxiety escalation—psychological exhaustion” vicious cycle under involution pressure, providing strong emotional resilience support for university students.

### 4.4 Psychological resilience and self-compassion as partial mediators between perception of involution and emotional exhaustion

This study found that PR partially mediated the relationship between POI and EE, supporting Hypothesis 4. This finding aligns with the research by Ala et al. (2024), who found that individuals with higher levels of PR demonstrated better emotional regulation and significantly lower levels of EE in the face of stress, based on a sample of 526 university students. According to COR, PR may relate to individuals' relative stability in psychological resources under stressful situations, greater adaptability and problem-solving abilities, and a lower likelihood of EE (Wang et al., 2021). Particularly in highly competitive, resource-constrained involution environments, PR may coincide with students' reframing of their perceptions of competition and less frequent negative emotional experiences related to involution. In this process, PR may serve as a psychological resource that is linked to lower emotional stress related to POI and lower levels of EE, showing a significant statistical mediating role (Jeong and Song, 2022). SC also partially mediated the relationship between POI and EE, further supporting Hypothesis 4. This finding is consistent with the research by Kotera et al. (2022), which found that individuals with higher levels of SC are more likely to approach stress and setbacks with acceptance, care, and a non-judgmental attitude, a pattern linked to less negative emotion and a lower likelihood of EE. Sharma et al. (2025) also pointed out that SC corresponds to less emotional distress in high-pressure environments and lower observed burnout incidence. Given the high similarity in stress mechanisms between burnout in the workplace and EE in involution contexts, this research strongly supports the mediating role of SC in the present study model. In this process, SC acts as a bridge and may reflect individuals' gentler and more rational responses to involution pressure, less self-criticism and excessive emotional depletion, and a weaker POI–EE association. From a positive psychology perspective, PR and SC, as two positive psychological traits, collectively showed statistical mediating roles in this study, with higher PR and SC linked to more favorable psychological adjustment in involution contexts and a lower likelihood of EE (Anjum et al., 2022; Choi et al., 2018). University students with higher levels of PR and SC are more likely to cope with stress in a positive way, which may help buffer emotional reactions related to social comparison and failure experiences, and is consistent with a weaker association between POI and EE.

### 4.5 Group differences

The group analysis results showed that females scored higher than males on POI, EE, and PR, while males scored higher on SC. Gender differences may be related to differences in emotional coping strategies and societal role expectations: females are more likely to experience anxiety in competitive situations and tend to use emotion-focused coping strategies (e.g., ruminating, worrying). While this may be associated with emotional expression and the acquisition of social support, it may also coexist with greater emotional burdens and feelings of exhaustion (Nolen-Hoeksema, 2000). At the same time, this proactive help-seeking pattern may be consistent with resource replenishment and the higher levels of PR observed in females (Tamres et al., 2002). In contrast, males, in the context of traditional gender norms, are more likely to suppress emotions and bear stress alone, a pattern associated with more constrained development of PR but, when facing setbacks, may be relatively more self-accepting and show higher levels of SC (Tamres et al., 2002). Notably, the gender-related difference appeared most evident for POI, whereas the differences in EE, SC, and PR were relatively smaller. This pattern suggests that gender may be more closely linked to how students perceive competitive and comparative academic contexts than to broad differences in EE or psychological resources. Therefore, gender-sensitive support should not only focus on emotional symptoms, but also on students' subjective interpretation of involution-related competition.

Regarding academic-year differences, 4th-year students had the highest POI scores, while 3rd-year students had the lowest, reflecting the varying stress characteristics at different stages. 4th-year students face multiple challenges, such as employment and graduation, which may be associated with higher POI, whereas 3rd-year students have generally adapted to the academic rhythm. The academic-year pattern should also be interpreted with caution. Differences were observed for POI and PR, but not for EE or SC, suggesting that grade-related variation was more closely reflected in students' perceptions of involution and resilience resources than in EE or SC. This may indicate that academic stage is more relevant to students' perception of external competition and resilience-related resources than to all aspects of psychological adjustment (Yang et al., 2025).

The subgroup findings based on POI level add further nuance to these results. The high-POI group was characterized by a less favorable psychological profile, but this pattern was more apparent for EE and PR than for SC. This suggests that when students perceive stronger involution, EE and resilience-related resources may be more sensitive indicators of subgroup differences. Moreover, the different directions of the POI–PR association in the high- and low-POI groups imply that perceived involution may not have the same psychological meaning across POI levels. From the perspective of Study Demands–Resources theory, manageable competitive demands may coexist with relatively preserved psychological resources, whereas stronger involution-related perceptions may be linked to resource strain and exhaustion (Bakker and Mostert, 2024; Ni et al., 2024).

These results indicate significant differences in psychological responses to POI based on gender, academic year, and POI level, suggesting that future interventions should develop targeted support strategies based on group characteristics.

## 5 Implications

### 5.1 Theoretical implications

This study, combining the perspectives of COR and positive psychology, constructs a theoretical framework for understanding the association between POI and EE. First, the study supports the role of POI as a unique psychosocial stressor, indicating that it has a direct statistical link with higher EE in the context of resource depletion. This finding expands the traditional view of stress research by incorporating “involuntary competition” and “high investment—low return” into the EE model, providing theoretical support for subsequent measurement and intervention strategies.

Second, the study supports the statistically significant indirect roles of PR and SC in the relationship between POI and EE, highlighting the protective value of positive psychological resources in coping with stress. PR is linked to greater adaptability and less resource depletion, while SC is linked to lower emotional burdens, self-criticism, and negative comparisons. This finding enriches the application of COR in dynamic stress contexts and emphasizes the important role of positive psychology in emotional regulation.

Finally, multi-group analysis reveals group-level heterogeneity in the POI–PR association. The high-POI group showed a stronger absolute POI–PR path coefficient, and gender analysis further indicated meaningful differences in students' psychological responses across groups. These results provide new perspectives for understanding the heterogeneity of stress responses and offer theoretical guidance for future intervention designs.

### 5.2 Practical implications

Higher education institutions should develop differentiated mental health intervention programs based on gender and academic year characteristics. For female students, the focus should be on emotional management and SC training, with attention to psychological pressure related to excessive rumination. For male students, interventions should emphasize stress coping strategies and PR development, with the goal of supporting adaptability and coping abilities. At the academic year level, senior students (4th-year) face significant graduation and employment pressures and should be provided with career planning and emotional support, while 1st-year students should focus on life adaptation and psychological adjustment.

Regarding the content of interventions, universities can enhance students' understanding of the mechanisms behind POI and EE through mental health courses, cultivating positive cognitive patterns. By integrating stress management workshops, psychodrama, and other practical activities, universities can strengthen coping skills, helping students master scientific emotional regulation and stress management techniques to develop good psychological coping abilities.

PR and SC are important psychological resources relevant to lower EE and should be key focuses of interventions. Universities can design resilience training camps and SC cultivation programs, combining psychological counseling and practical training to improve students' stress resistance and self-acceptance levels. This may help reduce the risk of EE and support the development of mental health.

## 6 Limitations and future directions

First, in terms of research design, this study employed a cross-sectional survey method, which, while revealing correlations between variables, does not establish causal pathways. Future research could consider using longitudinal tracking designs or experimental intervention methods and collecting data at multiple time points to further examine the temporal ordering and potential mechanisms among variables. Second, in terms of measurement, this study primarily relied on self-reported questionnaires, which may be associated with social desirability bias and common method bias. Although Harman's one-factor test and procedural considerations suggested that common method bias was not a dominant concern in this dataset, this issue cannot be fully excluded because the main constructs were collected from the same respondents using the same survey format. Future studies could incorporate in-depth interviews, case studies, peer reports, behavioral indicators, or multi-wave data collection to obtain richer and more diversified evidence. Currently, this study focuses on the associative pathways linking POI with EE among university students, without considering the interactions between multiple stressors. Future research could broaden the perspective on stress and explore the differential associations of various stress factors with EE, which may help refine the theoretical model and improve the specificity of intervention strategies.

## 7 Conclusion

This study explored the relationship between POI and EE among university students and examined the mediating roles of PR and SC. Through a survey of 763 university students, the results revealed a significant positive correlation between POI and EE, with PR and SC playing partial mediating roles in this relationship. Gender and academic year differences were found in the main variables: females scored higher than males on POI, EE, and PR, while males scored higher on SC. Further group analysis indicated that the high-POI group exhibited more severe EE, lower levels of PR and SC, and a stronger absolute POI–PR path coefficient. This study enriches the theoretical understanding of psychological adaptation mechanisms in involution contexts and provides practical references for mental health interventions in higher education.

## Data Availability

The raw data supporting the conclusions of this article will be made available by the authors, without undue reservation.
