# Supplementary material for: The associational pathway of emotional exhaustion among college students under perception of involution: the mediation model of self-compassion and psychological resilience
Source: Front Psychol. 2026 Jun 23;17:1811047. doi: 10.3389/fpsyg.2026.1811047 (PMC13337769; doi:10.3389/fpsyg.2026.1811047)
Supplement: Supplementary file 1 [file Supplementary_file_1.docx]

Table S1. Discriminant Validity Assessment via HTMT Index

|  | POI | SC | EE | PR |
| --- | --- | --- | --- | --- |
| POI |  |  |  |  |
| SC | 0.247 |  |  |  |
| EE | 0.602 | 0.498 |  |  |
| PR | 0.260 | 0.129 | 0.155 |  |

Note: POI, Perception of involution; EE, Emotional exhaustion; SC, Self-compassion; PR, Psychological resilience.

Table S2. Fornell-Larcker Matrix for Testing Discriminant Validity

|  | POI | SC | EE | PR |
| --- | --- | --- | --- | --- |
| POI | ***0.766*** |  |  |  |
| SC | -0.235 | ***0.842*** |  |  |
| EE | 0.560 | -0.470 | ***0.927*** |  |
| PR | -0.253 | -0.114 | -0.154 | ***0.762*** |

Note: POI, Perception of involution; EE, Emotional exhaustion; SC, Self-compassion; PR, Psychological resilience; AVE, average variance extracted; The square root of the AVE value is displayed in bold and italic on the diagonal of the table.

Table S3. MICOM Step 2_Compositional Invariance: across High vs. Low

| Construct | Correlations among construct scores | 5% Quantile of Empirical Distribution of *c_u_* | *p* | Compositional Invariance? |
| --- | --- | --- | --- | --- |
| POI | 0.999 | 0.987 | 0.746 | Yes |
| SC | 0.999 | 0.998 | 0.328 | Yes |
| EE | 1.000 | 1.000 | 0.010 | No |
| PR | 0.993 | 0.989 | 0.149 | Yes |

Note: POI, Perception of involution; EE, Emotional exhaustion; SC, Self-compassion; PR, Psychological resilience.
